# Transcriptional repression of lncRNA and miRNA subsets mediated by LRF during erythropoiesis

**DOI:** 10.1007/s00109-023-02352-1

**Published:** 2023-07-24

**Authors:** Katerina Athanasopoulou, Vasiliki Chondrou, Panagiotis Xiropotamos, Georgios Psarias, Yiannis Vasilopoulos, Georgios K. Georgakilas, Argyro Sgourou

**Affiliations:** 1 Biology Laboratory, School of Science and Technology, Hellenic Open University, 26335 Patras, Greece; 2 Laboratory of Genetics, Section of Genetics, Cell Biology and Development, Department of Biology, University of Patras, 26504 Patras, Greece; 3 Laboratory of Hygiene and Epidemiology, Faculty of Medicine, University of Thessaly, 41222 Larisa, Greece

**Keywords:** LRF overexpression, Erythropoiesis induction, lncRNA expression, miRNA differential expression, CpG island methylation

## Abstract

**Abstract:**

Non-coding RNA (ncRNA) species, mainly long non-coding RNAs (lncRNAs) and microRNAs (miRNAs) have been currently imputed for lesser or greater involvement in human erythropoiesis. These RNA subsets operate within a complex circuit with other epigenetic components and transcription factors (TF) affecting chromatin remodeling during cell differentiation. Lymphoma/leukemia-related (LRF) TF exerts higher occupancy on DNA CpG rich sites and is implicated in several differentiation cell pathways and erythropoiesis among them and also directs the epigenetic regulation of hemoglobin transversion from fetal (HbF) to adult (HbA) form by intervening in the γ-globin gene repression. We intended to investigate LRF activity in the evolving landscape of cells’ commitment to the erythroid lineage and specifically during HbF to HbA transversion, to qualify this TF as potential repressor of lncRNAs and miRNAs. Transgenic human erythroleukemia cells, overexpressing LRF and further induced to erythropoiesis, were subjected to expression analysis in high LRF occupancy genetic loci-producing lncRNAs. LRF abundance in genetic loci transcribing for studied lncRNAs was determined by ChIP-Seq data analysis. qPCRs were performed to examine lncRNA expression status. Differentially expressed miRNA pre- and post-erythropoiesis induction were assessed by next-generation sequencing (NGS), and their promoter regions were charted. Expression levels of lncRNAs were correlated with DNA methylation status of flanked CpG islands, and contingent co-regulation of hosted miRNAs was considered. LRF-binding sites were overrepresented in LRF overexpressing cell clones during erythropoiesis induction and exerted a significant suppressive effect towards lncRNAs and miRNA collections. Based on present data interpretation, LRF’s multiplied binding capacity across genome is suggested to be transient and associated with higher levels of DNA methylation.

**Key messages:**

- During erythropoiesis, LRF displays extensive occupancy across genetic loci.
- LRF significantly represses subsets of lncRNAs and miRNAs during erythropoiesis.
- Promoter region CpG islands’ methylation levels affect lncRNA expression.
- MiRNAs embedded within lncRNA loci show differential regulation of expression.

**Supplementary Information:**

The online version contains supplementary material available at 10.1007/s00109-023-02352-1.

## Introduction

Regulation of gene expression based on mutual epigenetic mechanisms, mainly DNA methylation levels, differential transcription of ncRNA species, and recruitment of histone modifying and chromatin remodeling systems, is attracting research attention in recent years. Of particular interest is the regulation of ncRNA expression mediated by TFs imitating the classical protein coding genes’ transcriptional regulation mechanisms. Correct tissue-specific and time-defined production of miRNAs and lncRNAs, of the most studied ncRNA varieties, constitutes the endogenous chromatin signaling towards a synchronized gene expression regulation. Among other ncRNA-dependent cellular processes, human erythropoiesis and hemoglobin switch from embryonic to HbF and to HbA during gestation are orchestrated by specific sets of miRNAs, lncRNAs, and co-operation of TFs [1–4]. Gene expression modifying TFs and miRNAs targeting and affecting TFs’ concentration, as well as lncRNAs with chromatin modulatory activity and “sponge” functions against miRNAs, participate in a sequential process supervising the hemoglobin switches during human fetal ontogeny.

lncRNAs have long been implicated in human hematopoiesis by contributing to the conformation of an accessible chromatin landscape in a tissue and stage specific manner [5]. Cross-acting of lncRNAs with genome and/or DNA binding proteins suggests an essential role on gene’s expression regulation, which has been already documented in general [6, 7] and more strictly in hematopoietic stem cell differentiation [8]. *DANCR*-lncRNA (Differentiation Antagonizing Non-Protein Coding) promotes erythroid differentiation [4], whereas *MONC*- (Megakaryocytic Oncogenic Non-Coding RNA) and *MIR100HG*-lncRNAs reside within cell nucleus and are involved in the regulation of erythro-megakaryocytic development. Their abnormally high expression has been evaluated in acute megakaryoblastic leukemia (AMKL) [9]. Along erythropoiesis pathway, hemoglobin switch form HbF (α_2_γ_2_ chains) to HbA (α_2_β_2_ chains), which under normal conditions occurs at the latest stage of gestation before birth, has been combined with *BGLT3*-lncRNA (Beta Globin Locus Transcript 3) expression [2]. Besides, CpG islands abundance lengthwise lncRNAs’ loci are associated with Polycomb Repressive Complex (PRC) recruitment, which spread chromatin repressive histone marks by promoting histone and DNA methylation, thus regulating downstream gene expression with variable potency [10].

MiRNAs have been also reported to affect hemoglobin switch from HbF to HbA and in certain circumstances to alleviate anemia symptoms in β-hemoglobinopathies patients. Research efforts to reactivate HbF expression in adult β-hemoglobinopathies patients are ongoing with the ultimate goal of benefiting patients from anemia symptoms and support their de-addiction from constant blood transfusions. Repressing effects towards BCL11A (B-Cell CLL/Lymphoma 11A), the most fully studied γ-globin repressor, are exerted by miR-15a/16–1, miR-486-3p, and miR-210; therefore, HbF levels are increased with valuable results for the patients [1, 11, 12]. MiRNAs are also identified to affect other modifying TFs of the β-globin locus, such as miR-26b which targets GATA-1 (Globin Transcription Factor 1), with indirect up-regulating effects on HbF expression [13] and miR-223-3p associated with LMO2 (LIM Domain Only 2) factor, leading to down-regulation of HbF levels [14]. Complex networks involving miRNAs, lncRNAs, other nuclear ncRNA species and mRNAs are also in the frontline of research efforts. A network consisting of *UCA1*- and *ZEB1-AS1*-lnRNAs with miR-548j, miR-3646, miR-937, and miR-19b-3p is confirmed to regulate HbF [15], while overexpressed *NR_001589*- and *uc002fcj.1*-lncRNAs in a high-HbF patient group were significantly correlated by bioinformatics methodology with certain sets of miRNAs and mRNAs [16] exhibiting an integrated chromatin modulatory capacity as a cluster.

A yet unsolved issue is decoding the pathway directing the epigenetic elements to a favorable repertoire of action during healthy state or disorder. Genome architecture and epigenetic landscape support positively or negatively ncRNA transcription, depending on the conditions prevailing across genetic loci coding for miRNA clusters, lncRNAs, and other ncRNA species, not sufficiently studied. lncRNAs are considered mainly products of RNA polymerase II transcription [17], and several TFs have been proved to control lncRNA expression in a tissue-specific and high-selective manner. Deregulation of TFs’ expression and a consequent tissue-imbalanced lncRNA expression are often sufficient for the development of several human disorders and for malignant cell transformation [18]. Further to other identical characteristics with “ordinary” genes, such as splicing and polyadenylation, many genetic loci-transcribing lncRNAs include CpG islands with potential regulatory role in their expression levels. Also, many genetic loci of miRNA genes encompass CpG islands, and DNA methylation status regulates miRNA expression. Expression abnormalities in a cancer-specific fashion associated with DNA methylation dependency are reported in several tumors, such as miR-31 in breast cancer [19].

So far, CxxC zinc finger protein 1 (or CFP1) has been shown to bind CpGs associated with active transcription start sites and enhance gene expression [20]. Also, other TFs or subunits of complexes with enzymatic activity to chromatin, such as MLL1/2 (myeloid/lymphoid factor), KDM2A/B (lysine demethylase), and TET1 (ten-eleven translocation), have common zinc finger domains and are reported to interact with CpG islands in mammals [21]. LRF belongs to an evolutionarily conserved family of TFs with zinc finger domains and has been shown to have binding preference at CG-rich and CpG island containing promoters in mouse [22]. In humans, LRF among a variety of known functions [23], binds at the 5′ end of *BGLT3*, encoding for a lncRNA spanning the intergenic sequence between γ- and β-globin gene and simultaneously at hypersensitive sites of β-globin Locus Control Region (βLCR), supporting hemoglobin transversion from HbF to HbA [24].

To assess the expression levels of miRNAs and lncRNAs influenced by LRF and implicated in hemoglobin switch from HbF to HbA, we have developed an in vitro system of transgenic human erythroleukemia cell line K562, over-expressing LRF transcription factor. Natural process of hemoglobin switch in humans is under control of the βLCR and other *cis* and *trans* regulatory elements, mainly modifying TFs acting to the β-type globin locus [3, 25], with BCL11A and LRF recognized as the most dominant repressors of HbF; however, LRF’s aggregate of functions still remains elusive. K562 erythroleukemia cell line constitutively expresses HbF although emanated from an adult individual and thus is a well-suited model to study epigenetic mechanisms controlling erythroid commitment and hemoglobin switch form γ- to β-globin expression, which can be obtained upon cells’ treatment with hemin/erythropoietin cocktail. The aim of the present study is to investigate transcriptional regulation of lncRNAs and miRNAs attributed to TF LRF implicated in erythropoiesis and further to hemoglobin switch, leading to β-globin gene prominent inter-connection with LCR and thus favoring its expression against γ-globin.

## Materials and methods

### Cell culture, transfection, and erythroid induction

Human K562 erythroleukemia cells were cultured and transfected with episomal vector encompassing the coding sequence (exons 1 and 2) of *ZBTB7A* gene encoding for LRF, as previously described. Reporter gene eGFP has been included within the same transcriptional cassette, and long-term expression of the exogenous gene has been confirmed by fluorescence microscopy (LEICA Microsystems, Heerbrugg, Switzerland). LRF/*ZBTB7A* expression levels were evaluated and were up to 11-fold increased. In parallel, transfections were carried out with the vector backbone. Results obtained by qPCR analyses displayed insignificant differences versus untransfected K562 cells. Treatment of transfected and untransfected K562 cells with 30 μΜ hemin (AppliChem GmbH, Darmstadt, Germany) and 5 ng/mL erythropoietin (EPO) (Cell Signaling) for 3 h resulted to erythroid differentiation, hemoglobinization, and induction of β-globin expression [24] Genomic DNA and total RNA were extracted pre- and post-erythroid induction of K562 transfected and untransfected cell clones. Four different cell conditions were studied: K562 untransfected (UT), K562 UT post-hemin/EPO induction (UT+hemin/EPO), K562 transfected and overexpressing LRF (LRF-OE), and LRF-OE post-hemin/EPO induction (LRF-OE+hemin/EPO).

### ChIP-Seq data analysis

LRF-binding sites were identified by analyzing existing K562 UT, UT+hemin/EPO, LRF-OE, and LRF-OE+hemin/EPO cell samples from chromatin immunoprecipitation assays with antibodies against LRF (Gene Expression Omnibus accession ID GSE200135), combined with next-generation sequencing (NGS) of precipitated DNA fragments (ChIP-Seq), previously conducted by Chondrou et al. [24]. Reads were analyzed with FastQC (https://www.bioinformatics.babraham.ac.uk/projects/fastqc/) to assess quality. Minion [26] was also used to perform an exhaustive search for sequencing contaminants before and after applying Trimgalore (https://github.com/FelixKrueger/TrimGalore) to remove contaminants and low quality reads with parameters “-q 20–length 20.” The remaining reads were aligned to the human reference genome (GRCh38) with STAR v2.7.10a [27] using parameters “–alignIntronMax 1–outFilterMultimapNmax 1–alignEndsType EndToEnd–alignSJoverhangMin 9999–alignSJDBoverhangMin 9999.” Picard (*Picard Tools. Broad Institute. *http://broadinstitute.github.io/picard/) was subsequently applied to minimize the enrichment of PCR duplicates. Reads that were aligned to the mitochondrial genome were excluded from downstream analyses. LRF peaks were identified with MACS2 [28] with parameters “-g hs–call-summits–extsize 300-p 5e-2,” using the IgG/no-IP ChIP-Seq as background. Peaks from non-canonical chromosomes were removed. The analysis resulted in the characterization of 13,796 peaks in UT K562 cells, 40,166 in UT+hemin/EPO, 35,781 in LRF-OE, and 596,274 in LRF-OE+hemin/EPO.

### CpG methylation analysis with pyrosequencing

Pyrosequencing CpG assay was utilized to assess DNA methylation status across CpG islands located at 5′ prime ends of lncRNAs’ genetic loci. Genomic DNA was extracted from K562 cultivated cells (UT, UT+hemin/EPO, LRF-OE, and LRF-OE+hemin/EPO) with phenol:chloroform:isoamyl alcohol in 25:24:1 ratio (Sigma-Aldrich), according to standard protocol [29]. 1.5 μg of each isolated genomic DNA was bisulfite converted, PCR amplified, and sequenced with PyroMark^®^ technology (QIAGEN GmbH, Hilden, Germany), according to manufacturer’s instructions and as previously described [30]. PCR and sequencing primers are listed in Supplementary table 1.

### Expression profiles of lncRNAs with RT-qPCRs

Total RNA was extracted from K562 cultivated cells (UT, UT+hemin/EPO, LRF-OE and LRF-OE+hemin/EPO) using Trizol Reagent (Invitrogen Life Technologies, Germany) according to the manufacturer’s instructions. One microgram of total RNA was converted to cDNA by Quantitect Reverse transcription kit (QIAGEN GmbH, Hilden, Germany). lncRNA expression was assessed by qPCRs with sequence specific primer sets (Supplementary table 1), designed to support detection of the main transcripts of each lncRNA genetic loci. Reactions were performed in Rotor-Gene Q instrument (QIAGEN GmbH, Hilden, Germany) using the PowerTrack SYBRGreen Master Mix (Applied Biosystems). All reactions were run in triplicates in at least two independent experiments and normalized to *GAPDH* control gene. Undetectable expression levels were assigned to lncRNAs with Cq values above acceptable limits (above 32–33 PCR cycles). The 2^- ΔΔCt method or Pfaffl equation with PCR efficiency correction was used for data analysis [31, 32].

### Next-generation sequencing (NGS) of miRNAs and data analysis

Total RNA including microRNA species were purified from K562 cultivated cells (UT, UT+hemin/EPO, LRF-OE, and LRF-OE+hemin/EPO), and a library consisted of all four RNA samples was created with QIAseq miRNA library kit (QIAGEN GmbH, Hilden, Germany). As an initial step, adapters were ligated to the 3′ and 5′ ends of miRNAs, and subsequently, universal cDNA synthesis with UMI (Unique Molecular Indices) assignment, cDNA cleanup, library amplification, and library cleanup were performed. By this method, the presence of adapter dimers, the major contaminant in sequencing libraries, is eliminated. Downstream RNA sequencing reactions were performed in Illumina ISEQ (Illumina, Inc., US) instrument in single-ended mode with 75 bps and read length ranging from 1–1.5 million per sample, which is considered adequate, due to the short length of mature miRNAs (21–25 bp). The in-house generated miRNA-Seq samples from K562 UT, UT+hemin/EPO, LRF-OE, and LRF-OE+hemin/EPO cell clones were initially processed with custom Python scripts to remove reads that did not include the complete UMI sequence. Due to the distinctive size distribution of miRNA sequences and the FastQC quality assessment results, Trimgalore was used with parameters “-q 30–length 18.” Three alignment rounds were applied to account for the uniqueness of miRNA Biology related to the fact that some miRNA sequences originate from multiple genomic loci. Reads were initially aligned on mature miRNA sequences derived from miRBase v22.1 [33] using STAR with parameters “–PCR and sequencing primers are 1–alignIntronMax 1–alignEndsType EndToEnd.” Subsequently, reads that aligned with a mismatch on multiple miRNAs were re-aligned without allowing any mismatches. Reads that were flagged as unaligned in the two aforementioned alignment steps were re-aligned to the human reference genome (GRCh38) using STAR with parameters “–outFilterMismatchNmax 1–alignIntronMax 1–PCR and sequencing primers are Local.” Gencode v39 [34] and miRBase annotation files were combined with the uniquely mapped reads from the three alignment rounds to count reads overlapping with genes and miRNAs. Reads that aligned on miRNAs originating from multiple genomic regions were counted only once. Read counts were adjusted based on the identified UMI sequences, and genes/miRNAs with zero counts across all cell states were removed from subsequent analyses (Supplementary Table 2).

### Identification of cell-state-specific miRNA expression

DEGseq [35] was applied on miRNA read counts to identify differentially expressed miRNAs between LRF-OE+hemin/EPO and UT+hemin/EPO K562 cells, LRF-OE+hemin/EPO and LRF-OE, LRF-OE+hemin/EPO and UT, UT+hemin/EPO and UT, as well as LRF-OE and UT, respectively, with a log fold-change cutoff of 0.5 and an adjusted *p* value cutoff of 0.05 (Supplementary Table 3).

### lncRNA and miRNA gene promoter characterization

The transcription start site (TSS) of miRNA genes were downloaded from DIANA-miRGen v4 [36] repository that contains CAGE-Seq-derived TSSs as calculated by ADAPT-CAGE algorithm [37]. Promoter regions were defined by extending each TSS 1.5 kb upstream and 1 kb downstream. To achieve maximum sensitivity, the entire DIANA-miRGen set of miRNA gene TSSs was used which resulted in the definition of promoters in hundreds of tissues, cell lines, and primary cells. The promoters of differentially expressed miRNAs were intersected with LRF ChIP-Seq peaks derived from matching cell-states to narrow down the list of LRF:miRNA regulatory interactions.lncRNA TSSs were extracted from the Gencode v39 human annotation files, and promoter regions were calculated by extending TSSs 1.5 kb upstream and 1 kb downstream.

### Statistical analysis

Statistical analysis was performed with SPSS software version 20.0. One-way ANOVA with Tukey’s multiple comparisons post hoc tests or *t*-test was applied to compare expression levels between the different groups. *p* values < 0.05 was considered statistically significant.

## Results

### Selection of lncRNAs as LRF targets

lncRNAs selected for the present study met specific criteria; preserved LRF occupancy at promoter regions either co-occurring with CpG islands or not and/or functions related to erythropoiesis were already assigned to these molecules. To define LRF:lncRNA interactions upon hemin/EPO treatment, LRF peaks were divided into three groups using BEDTools intersect [38]: peaks unique to hemin/EPO-treated or untreated K562 cells and peaks found in both conditions. Peaks from the hemin/EPO-treated were annotated based on their overlap with lncRNA gene promoters. lncRNA promoters with overlapping LRF peaks, implying LRF occupancy during erythropoiesis induction, were selected for further study (Table 1).

**Table 1:** Chromosomal locations of lncRNAs, corresponding CpG islands and methylation levels of each CpG sequence analyzed

lncRNA gene	CpG island	Region analyzed	Methylation status
*** DANCR***	93	Chr4: 52,712,612–52,712,726	Variable < 10%, Fig. 2B
*** DLEU1/2***	101	Chr13: 50,081,550–50,081,674	Variable < 10%, Fig. 2B
*** ENSG00000225806***	342	Chr20: 58,889,759–58,889,717	76–79%
*** ENSG00000236140***	35	Chr1:145,287,637–145,287,513	47–53%
*** ENSG00000236617 (CHFR-DT)***	130	Chr12: 132,887,765–132,887,878	Variable < 10%, Fig. 2B
*** ENSG00000248925 (PDCD6-DT)***	93	Chr5: 271,529–271,413	Variable < 10%, Fig. 2B
*** ENSG00000249494 (DMXL1-DT)***	77	Chr5:119,071,449–119,071,342	Variable < 10%, Fig. 2B
*** ENSG00000254821***	53	Chr6:4,135,471–4,135,557	8–16%
*** ENSG00000263923***	102	Chr4: 98,928,906–98,929,026	Variable < 10%, Fig. 2B
*** ENSG00000267338***	175	Chr22:16,601,809–16,601,921	17–22%
*** ENSG00000273391***	157	Chr7:139,360,171–139,360,050	Variable < 10%, Fig. 2B
*** H19***	36	Chr11:1,996,329–1,996,211	54–64%
*** HOTAIRM1 upstream***	116	Chr7:27,095,663–27,095,791	56–60%
*** HOTAIRM1 downstream***	116	Chr7: 27,096,410–27,096,495	26–31%
*** MALAT1***	50	Chr11:65,497,584–65,497,709	Variable < 10%, Fig. 2B
*** MEG3***	45	Chr14:100,826,116–100,826,218	85–88%
*** MEG3***	78	Chr14:100,827,449–100,827,521	88–92%
*** NEAT1***	81	Chr11:65,422,373–65,422,492	Variable < 10%, Fig. 2B
*** PVT1***	94	Chr8:127,794,107–127,794,229	70–76%
*** ENSG00000249249***	-	-	-
*** ENSG00000271833***	-	-	-
*** ENSG00000287483***	-	-	-

Among selected lncRNAs, HOXA Antisense RNA (*HOTAIRM1*), Deleted In Lymphocytic Leukemia 1/2 (*DLEU1/2*), Maternally Expressed 3 (*MEG3*), Nuclear Paraspeckle Assembly Transcript 1 (*NEAT1*), Differentiation Antagonizing Non-Protein Coding RNA (*DANCR*), Plasmacytoma Variant Translocation 1 (*PVT1*), Metastasis-Associated Lung Adenocarcinoma Transcript 1 (*MALAT1*), and Imprinted Maternally Expressed Transcript *H19* are already associated with hematological disorders [39–47]. Additionally, long non-coding RNA transcript sequences, derived from *ENSG00000225806*, *ENSG00000236140*, *ENSG00000236617 (CHFR-DT)*, *ENSG00000248925 (PDCD6-DT)*, *ENSG00000249494 (DMXL1-DT)*, *ENSG00000254821*, *ENSG00000263923*, *ENSG00000267338*, and *ENSG00000273391* genetic loci, which are found in *Homo sapiens* and annotated by several databases (ENA, LncBook, Ensembl, LNCipedia, Ensembl/GENCODE, GeneCards), were selected for investigation as well. Publications related to these transcripts (when exist) refer to genome-wide surveys or analysis of RNA-seq data; however, any affiliated molecular processes are not yet evident. All above-mentioned lncRNA transcripts were flanked by CpG islands. Sequences and chromosomal locations of CpG islands were tracked from UCSC Genome browser (https://genome-euro.ucsc.edu/cgi-bin/hgGateway) (Table 1).lncRNAs exempted from neighboring CpG islands’ existence, but encompassing LRF peaks during erythropoiesis induction, were also subjected to expression analysis. *BGLT3*-lncRNA [2] and transcripts derived from *ENSG00000271833*, *ENSG00000249249*, and *ENSG00000287483* genetic loci were evaluated for the LRF influence within a CpG island-free context.

In total, 20 lncRNAs were studied for both methylation and expression levels. CpG island 116 of *HOTARLM1*-lncXRNA, due to its long sequence was sequenced with upstream and downstream sets of primers. Also, CpGs 45 and 78 were located upstream of *MEG3* loci and were both subjected to methylation analyses.

### LFR abundance and binding in methylated CpG islands during erythropoiesis induction

**Fig. 1:**
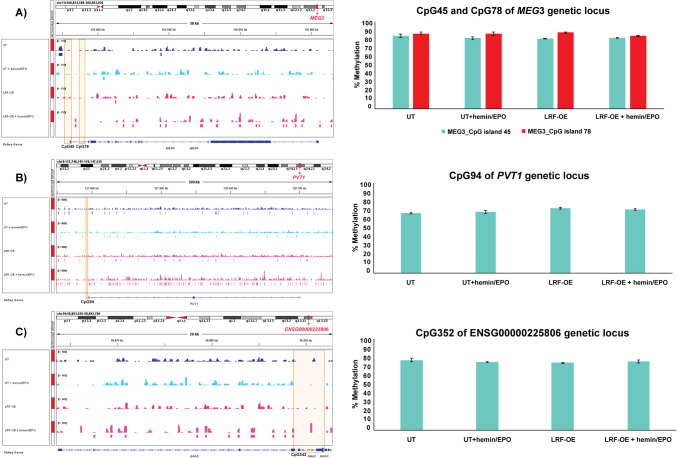
LRF occupancy sites at genetic loci of hypermethylated lncRNAs. *MEG* 3 (**A**), *PVT1* (**B**), and *ENSG00000225806* (**C**) displayed methylation levels between 70 and 92% both in UT and LRF-OE K562 clones pre- and post-hemin/EPO induction. LRF-binding sites were widely spread in all aforementioned K562 clones reinforcing the observation that methylation facilitates LRF binding. Methylation of whole region analyzed is presented as mean ± SE. ChIP seq data was retrieved from previous study [24]. CpG islands at 5' end of lncRNAs’ genetic loci are highlighted with orange squares

**Fig. 2:**
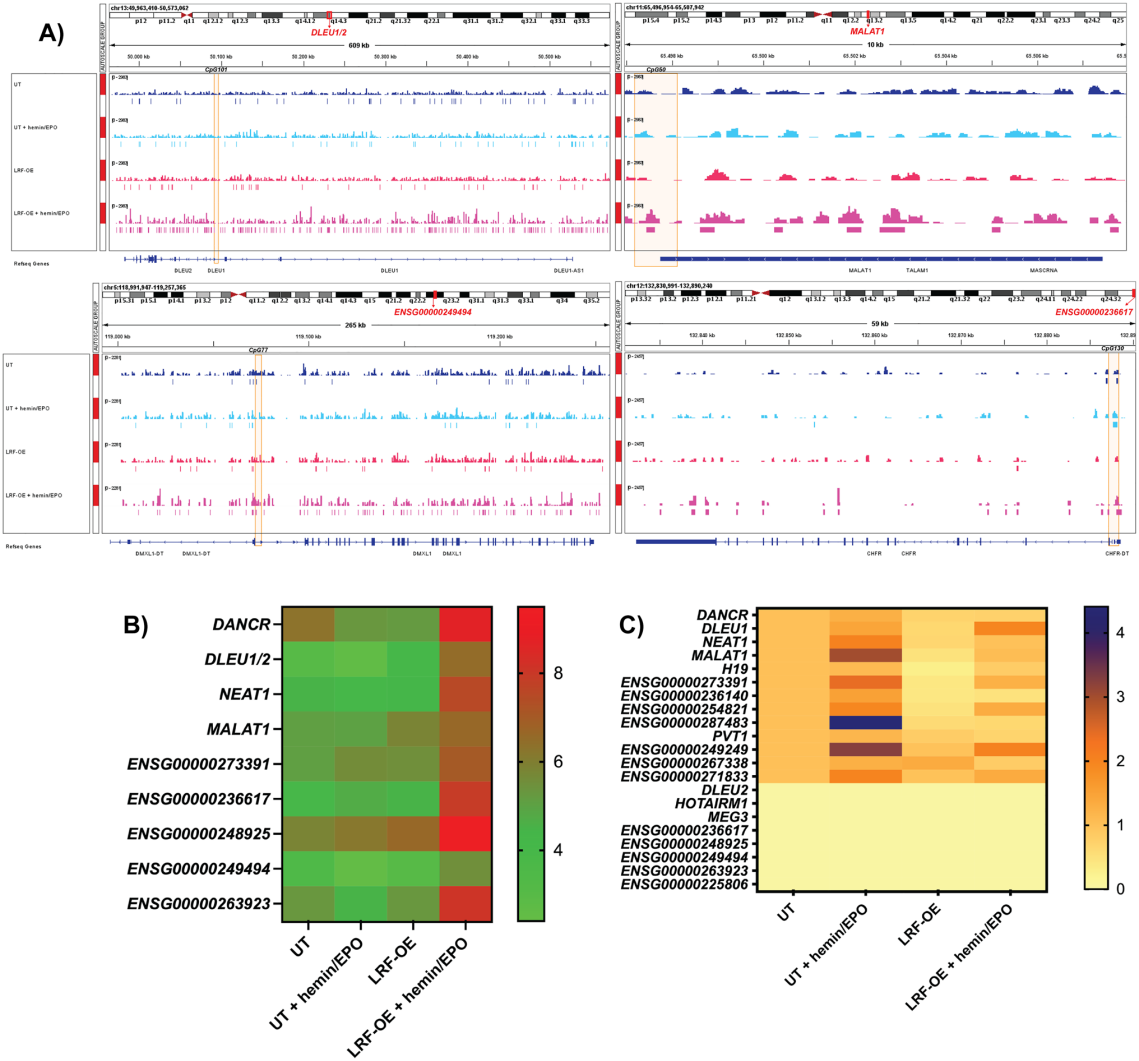
**A** LRF/*ZBTB7A* occupancy sites at lncRNAs genetic loci. Tracks display ChIP-seq data of LRF occupancy in untransfected (UT) and LRF-overexpressing (LRF-OE) K562 cells pre- and post-hemin/EPO induction. Calculated peak enrichment sites are depicted. LRF occupancy was ~ 43.2-fold more abundant in LRF-OE clones after erythropoiesis induction (LRF-OE+hemin/EPO). CpG islands at 5′ end of lncRNAs’ genetic loci are highlighted with orange squares. **B** Heatmap representing methylation status of CpG islands lying at promoter regions of lncRNAs and displaying medium to low methylation patterns. Methylation elevation is observed only in LRF-OE+hemin/EPO cell subgroup. Each square represents the mean percentage methylation of total sequence analyzed lengthwise each CpG island. A chromatic scale is shown on the right, in which red and green correspond to higher and lower methylation statuses, respectively. **C** Heatmap depicting expression levels of lncRNAs in UT and LRF-OE K562 cells pre- and post-erythropoiesis induction relative to UT non-induced cells. Cell subgroups of LRF-OE and LRF-OE+hemin/EPO display reduced expression levels of studied lncRNAs. A chromatic scale is shown on the right, in which blue and yellow correspond to a higher and lower expression respectively

LRF occupancy was more abundant in LRF-OE clones compared with UT (35,781 and 13,796 LRF peaks, respectively), whereas erythropoiesis induction with hemin/EPO treatment raised LRF affinity to DNA and peaks up to 5.7 × 10^5^ across genome (~ 43.2-fold increase compared to UT) (Figs. 1 and 2A). LRF responded to hemin/EPO treatment (within the 3-h induction) by increasing recognition and density of its binding sites across K562 cells’ genome, probably exerting its regulatory influence on primary and secondary gene loci during cell differentiation process towards the erythroid commitment.

CpG islands located 5′ upstream of lncRNA genetic loci were studied with pyrosequencing methylation assay. CpG island 342 of *ENSG00000225806*-lncRNA, both CpGs 45 and 78 of *MEG3*-lncRNA, and CpG 94 of *PVT1*-lncRNA were hypermethylated (methylation levels above 70% and up to 92%) in every single cytosine followed by guanosine deoxynucleotide with methylation potential (Fig. 1). CpG island 35 of *ENSG00000236140*-lncRNA, CpG island 36 of *H19*-lncRNA and upstream sequence of CpG 116 of *HOTAIRM1*-lncRNA displayed medium methylation status (between 50 and 60%) across CpG island’s sequence, whereas downstream sequence of CpG 116 as well as CpGs 175 and 53 of *ENSG00000267338*- and *ENSG00000254821*-lncRNAs, respectively, were methylated in low rate, between 16 and 26%. The rest examined lncRNA loci were hypomethylated with methylation mean values below 10% (Table 1). CpG sites with low to medium methylation status, mainly across CpGs 175, 116, 53, 36, and 35, showed high variance in methylation of single sites across the CpG sequence (Supplementary Fig. 1). *BGLT3-*, *ENSG00000287483*, *ENSG00000249249*, and *ENSG00000271833* genetic loci lacked CpG islands within 1.5 kb upstream of TSS and were examined only for lncRNAs’ expression levels.

A clear trend for increased methylation levels was obtained in LRF-OE+hemin/EPO cell clones lengthwise every CpG island sequence, whereas cell subgroups of UT, UT+hemin/EPO, and LRF-OE displayed insignificant levels of 5′ methyl-cytosines over unmethylated cytosines in corresponding CpG islands (Fig. 2B). Conclusively, aberrant expression levels of LRF and simultaneous erythropoiesis induction (LRF-OE+hemin/EPO condition) were associated with increased methylation status of CpG islands examined, which facilitated the wide spread of recognition and occupancy by LRF. This result implies the sensing capability and advanced binding of LRF in genomic sites with up-shifted methylation patterns.

### lncRNA expression levels correlated to DNA methylation status

Transcription efficacy of studied lncRNA genetic loci demonstrated three differentially expressed groups: those with undetectable expression, those that exhibited equal levels of expression regardless of different cell conditions, and those with decreased expression levels, apparent during LFR-OE and LRF-OE post-erythropoiesis induction. Correlations between DNA methylation status of CpG islands and expression levels led to the following results: transcription was undetectable in *ENSG00000225806*-, *HOTAIRM1*-, and *MEG3-*lncRNAs, derived from highly methylated genetic loci (above 60–70%), and analogous results were obtained from *DLEU2-*lncRNA and *ENSC00000236617-*, *ENSC00000248925-*, *ENSC00000249494-*, and *ENSC00000263923-*lncRNAs, comprising the group with hypomethylated CpG islands (bellow 10%) (Fig. 2C). *PVT1*-lncRNA, which was flanked by a hypermethylated CpG island (methylation levels above 70%) was found exceptionally active. Notably, *DLEU2-*lncRNA shares the same CpG island with *DLEU1*-lncRNA, an alternative transcript; however, they were differentially expressed in all experimental conditions of the present study.

**Fig. 3:**
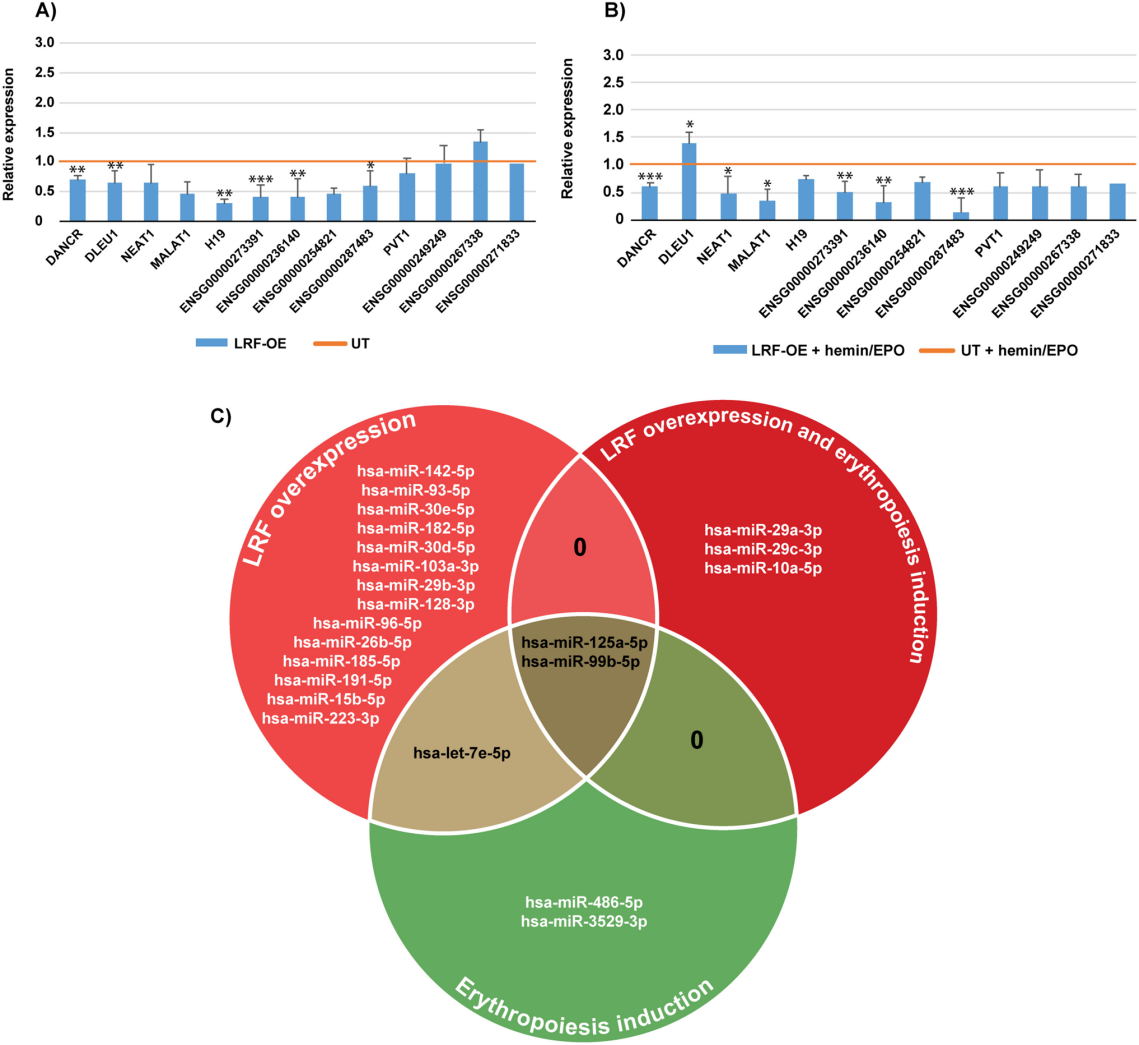
**A**, **B** Relative expression of lncRNAs in LRF overexpressing K562 clones before (LRF-OE) (**A**) and upon erythropoiesis’ induction (LRF-OE+hemin/EPO) (**B**). High levels of LRF reported lncRNAs’ expression. Data for *BGLT3*-lncRNA have been previously [24]. *p < 0.05, **p < 0.01, ***p < 0.001. **C** Venn diagram presenting common and unique differentially expressed miRNAs in cell sub-conditions. MiRNAs in red cycles are downregulated, those in green cycle are upregulated, and those highlighted in black are either downregulated when LRF was overexpressed or upregulated upon hemin/EPO treatment

Data interpretation of qPCRs particularly focused on cell subgroups of LRF-OE against the UT K562 cells and the LRF-OE with simultaneous induction of erythropoiesis against the corresponding UT subgroup (LRF-OE+hemin/EPO, against UT+hemin/EPO). LRF overexpression (LRF-OE) underscored its effect in *DANCR-*, *BGLT3-*, *DLEU1-*, *MALAT1-*, *NEAT1-*, *H19-*, *ENSG00000273391-*, *ENSG00000236140-*, *ENSG00000254821-*, and *ENSG00000287483*-lncRNAs, which were downregulated. Transcription levels of *DANCR-*, *BGLT3-*, *DLEU1-*, *H19-*, *ENSG00000273391-*, *ENSG00000236140-*, and *ENSG00000287483-*lncRNAs were decreased with significant values (*p* ≤ 0.001–0.05), compared to UT as reference (Fig. 3A). *ENSG00000249249-*, *ENSG00000267338-*, *ENSG00000271833-*, and *PVT1-*lncRNAs showed stable expression patterns under the same comparisons. These results highlighted the lncRNAs’ expression patterns when LRF is present in high levels within cells.

In LRF-OE+hemin/EPO cell clones, all mentioned lncRNAs (apart from the non-expressed and *DLEU1*-lncRNA) were more thoroughly repressed, and further, reduction of expression displayed by *DANCR-*, *BGLT3-*, *MALAT1-*, *NEAT1-*, *ENSG00000273391-*, *ENSG000002236140-*, and *ENSG00000287483*-lncRNAs was statistically significant compared to UT+hemin/EPO as reference (*p* ≤ 0.001–0.05) (Fig. 3B). Scores for CpG-free lncRNAs was 50% downregulation (*BGLT3-* and *ENSG00000287483*-lncRNAs) and 50% with invariant expression levels (*ENSG00000249249-* and *ENSG00000271833-*lncRNAs).

Conclusively, lncRNA expression inhibition in LRF-OE+hemin/EPO subgroup of cells was accompanied by methylation elevation in CpG islands located in lncRNA promoter regions and an extensive spreading of LRF occupancy sites across genomic regions.

### Down-regulation of miRNA expression mediated by LRF

Mature miRNAs (21–23 bp in length) differentially expressed between the four cell subgroups (UT, UT+hemin/EPO, LRF-OE, LRF-OE+hemin/EPO) were depicted by NGS and DEGseq [35] data analyses. Two miR clusters including miR182-5p and miR-125a-5p and miR99b-5p and let-7e-5p, respectively, simultaneously with single miRs, miR-486-5p, and miR3529-3p, were significantly upregulated upon erythropoiesis induction in both UT+hemin/EPO and LRF-OE+hemin/EPO, indicating the cell commitment to the erythroid lineage. These results are in consistency with other reported data [48]. MiR-126-5p was a unique case with repressed expression during erythropoiesis induction of UT with hemin/EPO treatment (Supplementary table 3). The top two overexpressed miRs, miR-19a-3p and mir16-5p with scores above 15 × 10^3^ reads (Supplementary table 2), were equally distributed in all cell subgroups.

LRF-OE cell clones exhibited only decreased expression levels for several miRNA sets. The same cluster of miR-125a-5p and miR99b-5p (upregulated during hemin/EPO treatment) was downregulated in LRF overexpressing cells. Also, miR-29a-3p, miR-29c-3p, and miR-10a-5p (member of miR cluster including also miR-182-5p and miR-183) were exclusively downregulated in LRF-OE treated with hemin/EPO. Between LRF-OE versus UT and LRF-OE+hemin/EPO versus UT+hemin/EPO, only downregulated miRNAs with significant values were displayed and are illustrated as Venn diagram (Fig. 3C). Table 2 represents sets of miRNAs downregulated with significant values (adjusted *p* value < 0.05 and log2FC > │0.5│) and total reads above 100. In consistency with results obtained from lncRNA expression profiles, miRs were systematically suppressed during high levels of LRF expression within transfected cells and some particular miR species prevailed upon erythropoiesis induction (miR-99b-5p and miR-125a-5p), whereas miR29a-3p, miR10a-5p, and miR29c-3p were apparent only in LRF-OE+hemin/EPO. The depicted miRNA sets from NGS followed by bioinformatics analysis are implicated in essential cell pathways presented in the “Discussion” section.

**Table 2:** Downregulated miRNAs (with significant values) in LRF-OE pre- and post-erythropoiesis induction

	LRF-OE vs. UT		LRF-OE + hemin/EPO vs. UT + hemin/EPO	
Gene name	log2(Fold_change)	Adjusted *p* value	log2(Fold_change)	Adjusted *p* value
hsa-miR-15b-5p	− 0.8	9.44E − 10	-	-
hsa-miR-29a-3p	-	-	− 0.8	0.01
hsa-miR-99b-5p	− 0.7	0.0010	− 0.5	0.0008
hsa-miR-125a-5p	− 0.7	1.15E − 15	− 0.5	3.11E − 28
hsa-miR-29b-3p	− 0.6	0.04	-	-
hsa-let-7e-5p	− 0.6	0.002	-	-
hsa-miR-185-5p	− 0.6	1.15E − 11	-	-
hsa-miR-142-5p	− 0.6	1.14E − 13	-	-
hsa-miR-223-3p	− 0.6	3.31E − 05	-	-
hsa-miR-128-3p	− 0.6	0.02	-	-
hsa-miR-93-5p	− 0.6	1.26E − 14	-	-
hsa-miR-182-5p	− 0.5	5.60E − 06	-	-
hsa-miR-30d-5p	− 0.5	0.01	-	-
hsa-miR-191-5p	− 0.5	1.38E − 08	-	-
hsa-miR-30e-5p	− 0.5	1.33E − 05	-	-
hsa-miR-96-5p	− 0.5	0.04	-	-
hsa-miR-103a-3p	− 0.5	0.0003	-	-
hsa-miR-26b-5p	− 0.5	0.0007	-	-
hsa-miR-10a-5p	-	-	− 0.5	1.92E − 06
hsa-miR-29c-3p	-	-	− 0.5	1.92E − 06

### lncRNA and miRNAs embedded in the same genetic loci are independently regulated despite common LRF occupancy

The inclusion of specific miRNAs within the same genetic loci by which the examined lncRNAs are transcribed was further investigated. Also, miRNA promoter regions that are either concurring or located at neighboring regions with lncRNA promoters were determined (Table 3) to map common genetic loci for miRNAs and lncRNAs and assess their potential co-regulation of expression mediated by LRF during erythropoiesis induction.

**Table 3:** Concurrent location of miRNAs and lncRNAs

lncRNA genetic loci	Encompassed MiRs	Encompassed MiR-promoters	Transcription direction lncRNAs/MiRs		LRF occupancy in MiR-promoters	MiRs from raw data (Supp. Table 2)
*** DANCR***		MiR–4449	+		LRF-OE+hemin/EPO	
*** DLEU1*** *** DLEU2***	MiR–3613	MIR-3613	+ -	-	LRF-OE+hemin/EPO	**3p**: UT, LRF-OE, LRF-OE+hemin/EPO **5p**: all cell subgroups
	MIR-15A	MIR-15A		-	UT+hemin/EPO, LRF-OE+hemin/EPO	**3p:** all cell subgroups **5p:** all cell subgroups
	MIR-16–1	MIR-16–1		-	UT+hemin/EPO, LRF-OE+hemin/EPO	**3p**: all cell subgroups
*** NEAT1***	MIR-612	MIR-612	+	+	LRF-OE+hemin/EPO	
*** MALAT1***			+			
*** HOTAIRM1***			+			
ENSG00000273391*	MIR-10399*					**3p**: UT, UT+hemin/EPO **5p**: LRF-OE+hemin/EPO
*** ENSG00000271833***			+			
*** ENSG00000249249***			+			
*** ENSG00000287483***			+			
*** ENSG00000225806***		MIR-296	+		UT+hemin/EPO, LRF-OE, LRF-OE+hemin/EPO	**3p**: UT, LRF-OE, UT+hemin/EPO, **5p**: all cell subgroups
		MIR-298				
*** MEG3***	MIR-770	MIR-770	+	+	UT+hemin/EPO, LRF-OE+hemin/EPO	
		MIR-732				
		MIR-136				**3p**: UT, UT+hemin/EPO, LRF-OE+hemin/EPO **5p**: UT, LRF-OE+hemin/EPO
		MIR-370				**3p**: UT
		MIR-493				**3p**: UT, UT+hemin/EPO, LRF-OE+hemin/EPO **5p**: all cell subgroups
		MIR-337				**3p**: LRF-OE **5p**: UT+hemin/EPO, LRF-OE, LRF-OE+hemin/EPO
		MIR-665				
		MIR-441				
		MIR-433				
		MIR-127				**3p**: all cell subgroups **5p**: UT, LRF-OE, LRF-OE+hemin/EPO
*** PVT1***	MIR-1204	MIR-1204	+	+	UT+hemin/EPO	
	MIR-1205	MIR-1205		+	LRF-OE+hemin/EPO	
	MIR-1206	MIR-1206		+	UT, LRF-OE, LRF-OE+hemin/EPO	
	MIR-1207	MIR-1207		+	LRF-OE, LRF-OE+hemin/EPO	
*** H19***	MIR-675		-	-		**5p**: UT
		MIR-4298			LRF-OE+hemin/EPO	
*** ENSG00000267338***			+			
ENSG00000236140**			**			
*** ENSG00000236617***			+			
*** ENSG00000248925***			-			
*** ENSG00000249494***			-			
ENSG00000263923**			**			
*** ENSG00000254821***			+			

^*^Encompassed in location of lncRNA genetic loci according to previous human genome assembly (GRCh37/hg19)

^**^Uncharacterized lncRNA genetic loci according to latest human genome assembly (GRCh38/hg38)

Promoter regions of miR-136, miR-493, miR-337, and miR-127, hosted within *MEG3*-lncRNA sequence, are detected in almost all cell subgroups; however, raw read counts of corresponding miRs are ≤ 10, implying insignificance as they are considered as ground noise (Supplementary table 2). Also, *MEG3*-lncRNA has a hypermethylated promoter and undetectable expression levels in all cell subgroups. Analogous results are displayed by miR-296, miR-10399, and miR-16–1 (with very low reads, ≤ 10) embedded in *ENSG00000225806*, *ENSG00000273391*, and *DLEU1/2* genetic loci respectively. Although these miRs show insignificant values of expression, the corresponding lncRNA hosts have demonstrated variable (detectable) levels of expression (Figs. 2C and 3A). On the contrary, miR-3613 and miR-15A, derived from *DLEU1/2* genetic locus and transcribed towards the same direction with *DLEU2*-lncRNA, which is silent in all cell subgroups, show high levels of expression. MiR-3613 displays 17–23 and miR-15A with 373–617 read counts among cell subgroups (UT, UT+hemin/EPO, LRF-OE, and LRF-OE+hemin/EPO) (Supplementary table 2).

Moreover, miR-1204, miR-1205, miR-1206, and miR-1207 were not disclosed among differentially expressed or detected miRNA species, whereas they are all located across *PVT1*-lncRNA genetic locus, found to be activated and expressed. Analogous results were obtained by promoter regions of miR-4449 encompassed within *DANCR*-lncRNA, miR-612 within *NEAT1*-lncRNA, and miR-4298 within *H19* genetic loci.

Results from lncRNAs’ genetic loci hosting miRNAs or their promoter regions suggest an independent mechanism underlying their expression regulation, although LRF occupancy is apparent across genetic locus, mainly in the LRF-OE+hemin/EPO subgroup. These results confirm certain studies that provide evidence for unconventional function between lncRNAs and encompassed miRNAs hosted in the same genetic loci [49], but further studies are necessary to relate the actual function of lncRNAs to their cognate miRNAs and potential cooperation of TFs regarding regulation of both ncRNA species expression.

## Discussion

Eukaryotic cells express selective fractions of the transcriptome, which defines the cell identity and phenotype [50]. Repression of tissue-nonspecific genomic loci is preserved by resident TFs, an effect that can be reversible or more stably down-regulated by general mechanisms of chromatin silencing, phenomena capable to persist through many rounds of cell division and differentiation. TFs’ affinity for DNA-binding sites integrates apart from the intrinsic DNA-binding preferences, interactions with other TFs, co-factors, and chromatin modifiers that are active in simultaneous local and temporal context [51], or affinity may vary depending on methylation patterns in CpG DNA sequences and modifications in histone marks [52].

LRF transcription factor, encoded from the human gene *ZBTB7A*, has been strongly connected with gene expression control and regulation of lineage fate decisions [53], influencing several branches of hematopoietic differentiation. It mainly represses Bim-mediated apoptosis and binds gene targets of the key erythroid transcription factor GATA1 [54] and also is implicated in γ-globin repression during the final hemoglobin switch to the adult form via a regulatory loop including up-regulation of *BGLT3*-lncRNA expression [24]. The present work originates from the idea that LRF, apart from protein genes, may also exert a regulatory action towards gene loci of non-coding RNA transcripts. Within this frame, 20 lncRNAs with pronounced LRF occupancy (Table 1) were investigated in terms of expression levels during induction of erythropoiesis (Fig. 3B) and hemoglobin switch in human K562 erythroleukemia cell line. Eight of these lncRNAs have already demonstrated an involvement in hematological disorders: *HOTAIRM1* gene is adjacent and antisense to the transcription start site of *HOXA1*, and its transcription is initiated from the shared promoter segment embedded in a CpG island between the two genes. *HOTAIRM1*-lncRNA is specifically expressed in the myeloid lineage, most highly in the terminal stage of granulocytic differentiation [39]. *DLEU1*-lncRNA, *DLEU2*-lncRNA, and a cluster of neighboring protein-coding tumor suppressor genes, located at 13q14.3, are frequently deregulated in chronic lymphocytic leukemia (CLL) [40]. Notably, miR-15a/16–1, which are embedded within the same locus with *DLEU1/2* gene, are expressed with a distinct mode [49] and also confirmed by our analysis. *MEG3*-lncRNA expression has been suggested as a potential biomarker for therapy response and survival profile in childhood acute lymphoblastic leukemia (cALL) [41], whereas encompasses miRNAs and promoter regions of miRNA species (Table 3). *NEAT1*-lncRNA is upregulated in multiple myeloma and it is involved in mechanisms of cellular stress response [42]. *DANCR*-lncRNA, apart from its role to erythroid differentiation [4], positively regulates acute myeloid leukemia chemoresistance to cytarabine by promoting autophagy of AML treated cells and acting as a sponge to decrease miRs abundance that target components of the autophagy machinery [43]. *PVT1* maps at 8q24 encodes linear and circular transcript isoforms and encompasses miRNAs (Table 3). *PVT1* genomic locus is downstream to *MYC*, and co-interactions have been described among these two genes. The role of *PVT1*-lncRNA in hematological malignancies highlights its involvement in disease progression and regulation of immune responses [44]. *Malat1*-lncRNA depletion in mice (highly conserved sequence with humans) inhibits the erythroid myeloid lymphoid cell proliferation [45]. In humans, *MALAT1*-lncRNA is recognized as a biomarker for atherosclerosis [46]. Reduced expression of *H19* Imprinted Maternally Expressed Transcript has been observed in chronic myeloid leukemia (CML), and myelomonocytic leukemia (CMML), as well as in AML [47], and is also the precursor of miR-675 known to downregulate the retinoblastoma (RB) gene, a fact that multiplies the level of complexity to its functional roles [55]. In the present study, lncRNAs hosting miRNAs or their promoter regions revealed differential expression patterns underlying the distinct regulatory mechanisms guiding their expression.

Additional lncRNA transcripts with apparent LRF peaks covering their promoter regions were also subjected to expression analysis although their roles in chromatin regulation have not been uncovered yet. Seventeen out of 20 lncRNAs examined were flanked by CpG islands either over-methylated and thus preventing transcript expression (apart from *PVT1*-lncRNA) (Fig. 1) or exhibiting variance in methylation patterns. A trend for increased presence of 5′ methyl-cytosines within CpG islands lying in lncRNA promoter regions was clearly demonstrated by LRF-OE+hemin/EPO cell subgroup (Fig. 2B, Supplementary Fig. 1). lncRNA expression repression was observed mainly in LRF-OE+hemin/EPO subgroup accompanied by increased methylation patterns and a simultaneous intense expansion of the LRF recognition sites lengthwise genomic loci investigated (Figs. 1 and 2A). Across promoters of miRNA species detected to be differentially expressed during LRF-OE and LRF-OE differentiating to the erythroid lineage with hemin/EPO treatment (Table 2), LRF showed increased binding affinity (Supplementary Fig. 2), in consistency with results emanated from lncRNAs. These results suggest the LRF capability to trace secondary binding sites that become visible when methylation levels of DNA are increased.

LRF-binding amplitude shown particularly in cell subgroup LRF-OE+hemin/EPO can be also explained by the availability of alternate direct binding partners. LRF belongs to ZBTB transcription factors family consisting of consensus zinc finger DNA-binding motifs and a BTB domain by which form obligate dimers [56]. Dimerization likely facilitates target DNA binding through the C-terminal zinc finger motifs, but additionally to mediating homodimerization, the BTB dimer forms a favorable scaffold for other ligands. High-throughput assays have revealed cooperative binding interactions at heterodimeric motifs, in some cases completely novel that modify the transcriptional regulation of target genes. The heterodimeric motifs can often be distinct from the individual TF’s in vitro binding preferences and may facilitate binding of TFs to weaker binding motifs [57]. LRF has already demonstrated its action as transcriptional repressor, with histone‐modifying complexes’ recruitment capability, that sequentially package the genetic locus transforming chromatin to non-accessible state [58]. These events prevent genes (including genes encoding for non-protein transcripts) from being transcribed and form the basis of stable epigenetic programs controlling gene loci expression potentially throughout differentiation processes, such as erythropoiesis induction.

As established by miRNA profiling across cell subgroups, many of the upregulated miRNAs expressed only when cells were treated with hemin/EPO to induce cell commitment to the red lineage play characteristic roles during hemopoiesis, such as miR cluster 125a/99b/let-7e and miR-10a and miR-29 family, which promote the hematopoietic stem cell (HSC) self-renewal and also have a prominent attribute to discrete hematopoiesis stages [48, 59]. These sets were all significantly repressed in the LRF overexpressing mode and the phenomenon intensified during terminal differentiation of K562 cells by erythropoiesis induction (Fig. 3B and C).

In cell subgroups of LRF-OE and LRF-OE+hemin/EPO, only significantly downregulated miRNA species were obtained (Table 2). MiR-15b-5p has been reported to function as both oncogene and tumor suppressor in several human cancers, while is sponged by lncRNAs, *PVT1*- and *MALAT1*-lncRNAs among them [60]. In the present study, miR-15b-5p was reduced, relevant to lncRNA repressed expression. Deregulation of miR29b-3p, which targets BCL11A transcription factor, is in the same direction with the observation that, during K562 differentiation, the expression of γ-globin decreases (mediated by BCL11A enhancement) with an anti-diametric increase of β-globin expression [11, 24, 61]. MiR-29b, miR-29a, and miR-29c are generated from two primary transcripts and compose the miR-29 family, located in chromosomes 7q32.3 and 1q32.2, respectively. They are highly conserved in mammals, and members of the family are implicated in human disorders such as osteoarthritis, osteoporosis, cardiorenal, and immune disease [62]. MiR-26b-5p, miR-185-5p, and miR-191-5p in peripheral blood mononuclear cells target genes related to the myo-inositol metabolism [63]. Pathway analysis (mirPath v.3, via DIANATOOLS) highlighted various signal transduction pathways that were associated to miR-142-5p, including TGF-β, signaling mediated by MAPK, the insulin/IGF pathway, as well as Erb and mTOR signaling [64]. MiR30e-5p expression is suppressed by IFN-α, a pivotal and abundant pro-inflammatory cytokine, and miR-223-3p has been assigned as a signature of response to anti-TNF-α therapy (TNF: tumor necrosis factor) [65, 66]. MiR-128-3p has been identified to target ZC3H12D, a member of the CCCH-type zinc finger-containing proteins family, which negatively regulates Toll-like receptor signaling and suppresses inflammatory cytokines as well as NF-κB [67, 68]. MiR-93-5p has been associated with inhibition of the epithelial protein lost in neoplasm (EPLIN) expression, a protein involved in regulating actin dynamics and cell mobility, thus has been proposed as novel therapeutic factor for inhibiting cancer angiogenesis and progression [69]. Conclusively, miRNA species found significantly repressed by LRF are implicated in myo-inositol metabolism [63], TGF-β, MAPK, insulin/IGF, Erb, and mTOR signaling pathway [64] expression of inflammatory cytokines [65, 66] and targeting transcription factors essential for several cell functions [67–69] with an indirect potential towards the erythropoiesis process.

## Conclusions

This study provided clear evidence for the repressing capability of LRF in ncRNA molecules, mainly lncRNAs and sets of miRNAs. lncRNAs exhibited differential methylation and expression patterns during LRF overexpression status and further, during erythropoiesis terminal differentiation with concurrent hemoglobin switch form γ- to β-globin. Also, specific miRNA species were significantly downregulated, as yet, remains to be elucidated the actual role of this network of non-coding RNAs towards the last stage of hemopoiesis and simultaneous transition from fetal to adult stage of hemoglobin production, in human non-malignant tissue-specific cell lines. Gene silencing is not necessarily dependent on the continuous residence of a sequence‐specific repressor at a control element. Assays that detect genome‐wide TF‐binding activity, such as ChIP‐seq typically, provide a static snapshot of occupancy precisely at the time point of the assay; however, LRF potentially exerts transcriptional repression based in DNA tethering by a fleeting way.

## Supplementary Information

Below is the link to the electronic supplementary material.

Supplementary file1 (DOCX 42 KB)

Supplementary file2 (DOCX 21 KB)

Supplementary file3 (DOCX 710 KB)

Supplementary file4 (DOCX 16 KB)

## Data Availability

Data generated in the present study is included in supplementary material.

## Abbreviations

*AMKL*: Acute megakaryoblastic leukemia
*AML*: Acute myeloid leukemia
*BCL11A*: B-cell CLL/Lymphoma 11A
*BGLT3*: Beta Globin Locus Transcript 3
*cALL*: Childhood Acute lymphoblastic leukemia
*CFP1*: CxxC zinc finger protein 1
*CHFR-DT*: CHFR divergent transcript
*ChIP-Seq*: Chromatin immunoprecipitation sequencing
*CLL*: Chronic lymphocytic leukemia
*CML*: Chronic myeloid leukemia
*CMML*: Chronic myelomonocytic leukemia
*DANCR*: Differentiation Antagonizing Non-Protein Coding RNA
*DLEU1*/2: Deleted in Lymphocytic Leukemia 1/2
*DMXL1-DT*: DMXL1 divergent transcript
*eGFP*: Enhanced green fluorescent protein
*EPLIN*: Epithelial protein lost in neoplasm
*EPO*: Erythropoietin
*GAPDH*: Glyceraldehyde-3-phosphate dehydrogenase
*GATA-1*: Globin Transcription Factor 1
*H19*: Imprinted Maternally Expressed Transcript H19
*HbA*: Adult hemoglobin (hemoglobin A)
*HbF*: Fetal hemoglobin (hemoglobin F)
*HOTAIRM1*: HOXA antisense RNA
*HSC*: Hematopoietic stem cell
*IFN-α*: Interferon alpha
*IGF*: Insulin-like growth factor
*KDM2A/B*: Lysine-specific demethylase
*LMO2*: LIM Domain Only 2
*lncRNA*: Long non-coding RNA
*LRF*: Leukemia-related factor
*LRF-OE*: K562 transfected and overexpressing LRF
*LRF-OE* + *hemin/EPO*: LRF overexpressing post hemin/EPO induction
*MALAT1*: Metastasis-Associated Lung Adenocarcinoma Transcript 1
*MAPK*: Mitogen-activated protein kinase
*MEG3*: Maternally expressed 3
*miRNA*: Micro RNA
*MLL1/2*: Myeloid/lymphoid factor
*MONC*: Megakaryocytic oncogenis non-coding
*mRNA*: Messenger RNA
*mTOR*: Mammalian target of rapamycin
*ncRNA*: Non-coding RNA
*NEAT1*: Nuclear Paraspeckle Assembly Transcript 1
*NF-κB*: Nuclear factor kappa B
*NGS*: Next-generation sequencing
*PDCD6-DT*: PDCD6 divergent transcript
*PRC*: Polycomb repressive complex
*PVT1*: Plasmacytoma Variant Translocation 1
*RB*: Retinoblastoma
*TET1*: Ten-eleven translocation
*TF*: Translation factor
*TGF-β*: Transforming growth factor beta
*TNF*: Tumor necrosis factor
*TSS*: Transcription start site
*UCA1*: Urothelial cancer associated 1
*UMI*: Unique Molecular Indices
*UT*: K562 untransfected
*UT*+*hemin/EPO*: K562 untransfected post hemin/EPO induction
*ZBTB7A*: Zinc finger and BTB domain-containing 7A
*ZC3H12D*: Zinc finger CCCH-type containing 12D
*ZEB1-AS1*: ZEB1 antisense 1
*βLCR*: β-Globin locus control region
