# Supplementary material for: Transcriptional repression of lncRNA and miRNA subsets mediated by LRF during erythropoiesis
Source: J Mol Med (Berl). 2023 Jul 24;101(9):1097–112. doi: 10.1007/s00109-023-02352-1 (PMC10482784; doi:10.1007/s00109-023-02352-1)
Supplement: Supplementary file 3 — Supplementary file3 (DOCX 710 KB) [file 109_2023_2352_MOESM3_ESM.docx]

**Supplementary figure 1**

LncRNAs displaying variable methylation pattern in single CpG sites lengthwise the studied CpG islands’ sequence. **(A)** CpG 35 of *ENSG00000236140* genetic locus **(B)** CpG 36 of *H19* genetic locus **(C)** CpG 116 upstream sequence of *HOTAIRM1* genetic locus **(D)** CpG 116 downstream sequence of *HOTAIRM1* genetic locus **(E)** CpG 175 of *ENSG00000267338* genetic locus and **(F)** CpG 53 of *ENSG00000254821* genetic locus. Methylation data are presented as mean percentage ± SE.

**Supplementary figure 2**

LRF/*ZBTB7A* occupancy sites at promoters of miRNAs downregulated in LRF-OE pre- and post- erythropoiesis induction. Tracks display peak enrichment sites for UT and LRF-OE cells pre- and post- hemin/EPO treatment. LRF binding affinity was more abundant in LRF-OE cells after erythropoiesis induction.

**
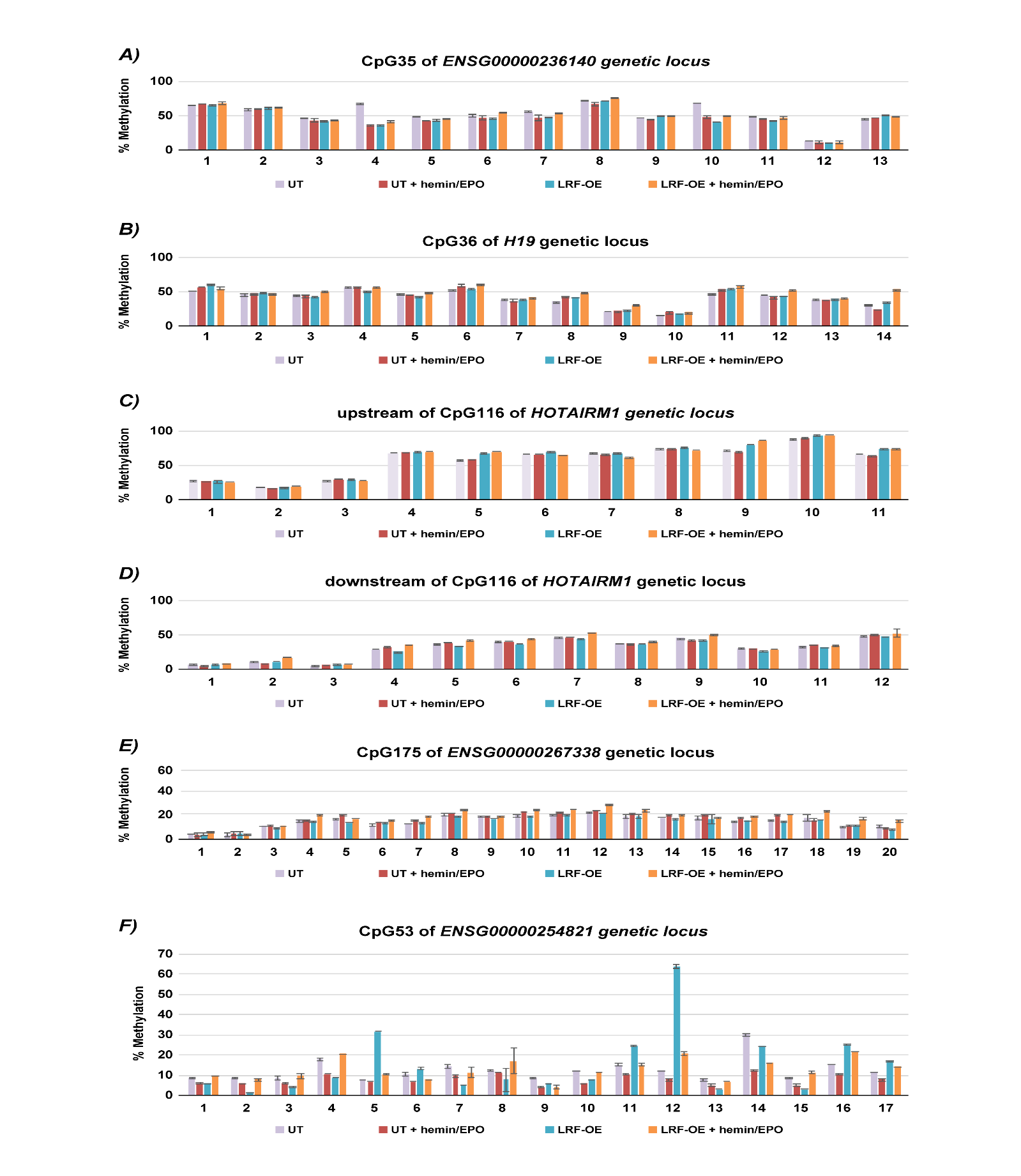
Supplementary figure 1**

**
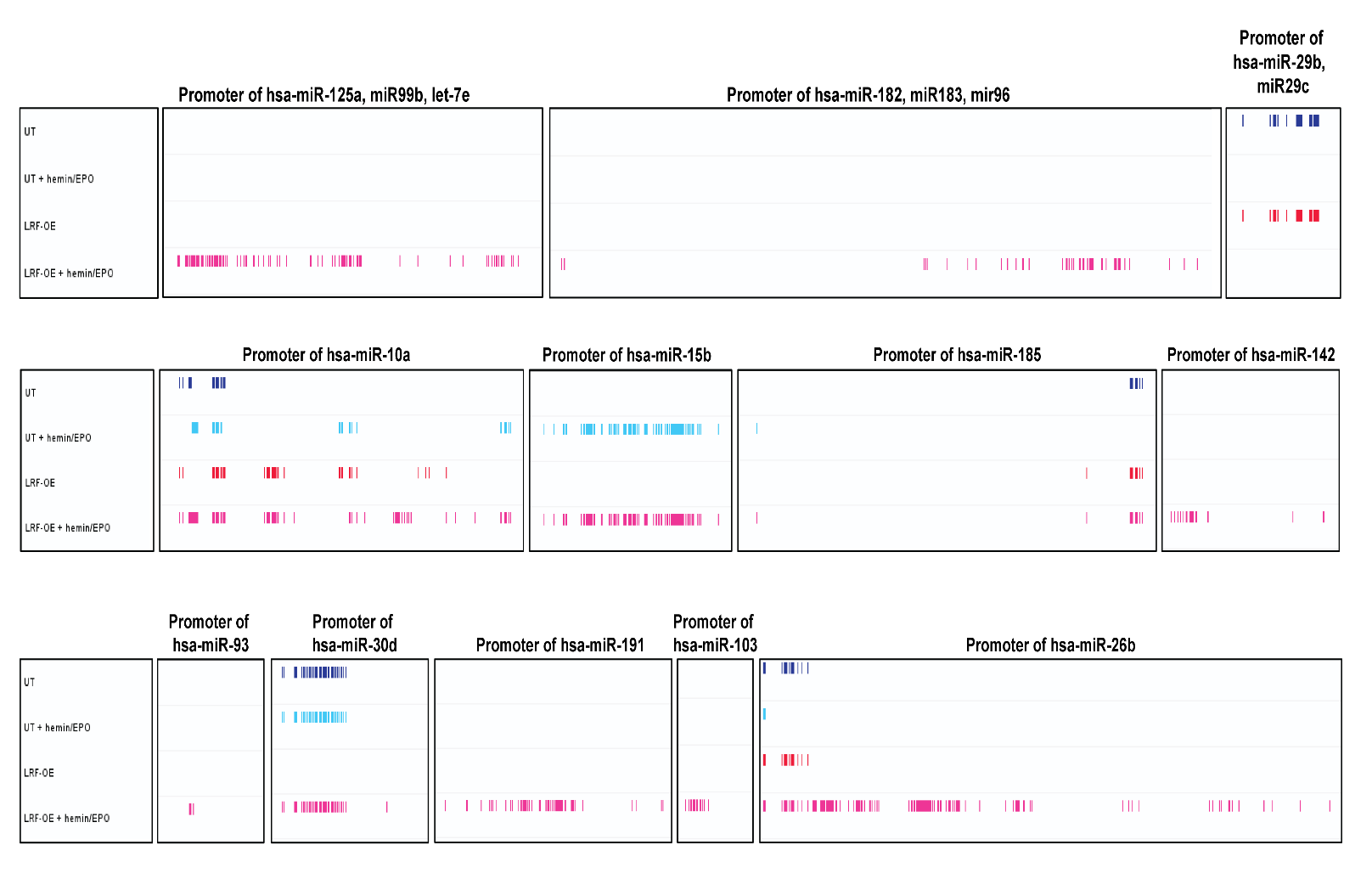
Supplementary figure 2**
