# Supplementary material for: Transcriptional repression of lncRNA and miRNA subsets mediated by LRF during erythropoiesis
Source: J Mol Med (Berl). 2023 Jul 24;101(9):1097–112. doi: 10.1007/s00109-023-02352-1 (PMC10482784; doi:10.1007/s00109-023-02352-1)
Supplement: Supplementary file 4 — Supplementary file4 (DOCX 16 KB) [file 109_2023_2352_MOESM4_ESM.docx]

**Supplementary table 1: Primer sets for qPCRs and Pyrosequencing (CpG) assays**

| Assay | lncRNA gene | Sequence | Primer |
| --- | --- | --- | --- |
| CpG assay | *DANCR* | 5'TAGGTTTTTTTGTTAGTTGGAGTTG 3' | Forward |
|  |  | 5' ACCTACCCTAAAAACTAAAACAACTACACTT 3' | 5’ biotinylated reverse |
|  |  | 5' GTGGGTTAGATTAGGGG 3' | Forward/  Pyrosequencing |
|  | *DLEU1/2* | 5' GGGGGGTTTTGTTGATAGAT 3' | Forward |
|  |  | 5' CAAATCTACTCTCACTTAATCCCCAAAAATCC 3' | 5’ biotinylated reverse |
|  |  | 5' GGGGTTTTGTTGATAGATT 3' | Forward/  Pyrosequencing |
|  | *ENSG00000225806* | 5' TGAGTTTTATGGTTATTTTTAGTATGG 3' | Forward |
|  |  | 5' AACCCCTAAAAATAAACAAACTCCTACCAT 3' | 5’ biotinylated reverse |
|  |  | 5' GTTTGGTTAGGGTTTTTTG 3' | Forward/  Pyrosequencing |
|  | *ENSG00000236140* | 5' GGTAGGAGGATTGAGTAAGGT 3' | Forward |
|  |  | 5' AAAACACAAACTCCCTCACTA 3' | 5’ biotinylated reverse |
|  |  | 5' TGGAAAATTGGAGAGATTGG 3' | Forward/  Pyrosequencing |
|  | *ENSG00000236617 (CHFR-DT)* | 5' GGGGAGGGGTTAGAGGTTT 3' | Forward |
|  |  | 5' AAAAAATCCCCACCTACACTACCTC 3' | 5’ biotinylated reverse |
|  |  | 5' GGTTATTTTTGATTTTGATTAG 3' | Forward/  Pyrosequencing |
|  | *ENSG00000248925*  *(PDCD6-DT)* | 5' TGTAGGGGGTAGGGTTTGGTATT 3' | Forward |
|  |  | 5' TTCCCACTATTAACAAATCACATTCACC 3' | 5’ biotinylated reverse |
|  |  | 5' TAGTAGTTAAGTTGTTAGGTA 3' | Forward/  Pyrosequencing |
|  | *ENSG00000249494*  *(DMXL1-DT)* | 5' GGTTAGTATTTGGTGTAGGTTTATG 3' | Forward |
|  |  | 5' CCCTAAACTTCACCTAAACTAAC 3' | 5’ biotinylated reverse |
|  |  | 5' GGTGGAGAAGGTAGAAGTA 3' | Forward/  Pyrosequencing |
|  | *ENSG00000254821* | 5' GTTGTTATTTAGATAATAGGGAAGGAG 3' | Forward |
|  |  | 5' TACCCCAAACTCCTACCTAAACTCT 3' | 5’ biotinylated reverse |
|  |  | 5' GGAAGGAGGGTTTTTTA 3' | Forward/  Pyrosequencing |
|  | *ENSG00000263923* | 5' AGGGAGGGAAAGGTTGTT 3' | Forward |
|  |  | 5' TATTACCTTTAACCCCCACCC 3' | 5’ biotinylated reverse |
|  |  | 5' GGGTTTTTATTAGAAAGTGT 3' | Forward/  Pyrosequencing |
|  | *ENSG00000267338* | 5' GTTGGAGTAGTTGTTAAAATTT 3' | Forward |
|  |  | 5' CTAATCACCAAAAAAAACACATAAATC 3' | 5’ biotinylated reverse |
|  |  | 5' GGTTAGGGGGAGGGGT 3' | Forward/  Pyrosequencing |
|  | *ENSG00000273391* | 5' TTTTGGGTTGGGGGATAAG 3' | Forward |
|  |  | 5' CCAAAACCCTTAAAATAAAACTCCACTC 3' | 5’ biotinylated reverse |
|  |  | 5' GGGATGGGGGAAGGT 3' | Forward/  Pyrosequencing |
|  | *H19* | 5' GGGGTGGAAGTGTTTATTAGTTGT 3' | Forward |
|  |  | 5' AAACCCCTCACCTTTCATATTATAAATTCT 3' | 5’ biotinylated reverse |
|  |  | 5' GGAAGTGATAAGTAGGATATGATAT 3' | Forward/  Pyrosequencing |
|  | *HOTAIRM1*  *Downstream CpG116* | 5' AGAGGTGAGAAGGGAAGAGG 3' | Forward |
|  |  | 5' CACCATCTCCCAACCCAACCTCAAA 3' | 5’ biotinylated reverse |
|  |  | 5' GAAGGGAAGAGGGTT 3' | Forward/  Pyrosequencing |
|  | *HOTAIRM1*  *Upstream CpG116* | 5' TTGGATTATAATTTGAGTGGGAGTAGGAT 3' | Forward |
|  |  | 5' CTCCTTCCTAAAATACCCCATACT 3' | 5’ biotinylated reverse |
|  |  | 5' AGTAGGATATTTTTAGGTTT 3' | Forward/  Pyrosequencing |
|  | *MALAT1* | 5' TGGGAAAGGAAGATTTAGATTGAAAATG 3' | Forward |
|  |  | 5' ACCCCAATCCTTTACAAAAATC 3' | 5’ biotinylated reverse |
|  |  | 5' AGAGTAGGTTAGAATTAGTGG 3' | Forward/  Pyrosequencing |
|  | *MEG3_45* | 5' GGGTGAGGTTAGAGTAATTTGTTATA 3' | Forward |
|  |  | 5' CCCATCCCAAAAACCAATACCCTCTTCCT 3' | 5’ biotinylated reverse |
|  |  | 5' ATATAAATTTTATTTAGTTAGTTT 3' | Forward/  Pyrosequencing |
|  | *MEG3_78* | 5' GGAGGTGGTTTTTGTTTTTTTGATGG 3' | Forward |
|  |  | 5' AACCTCTCTCTCCATCCTACTCA 3' | 5’ biotinylated reverse |
|  |  | 5' AGGGAATAGTTTTGAGATT 3' | Forward/  Pyrosequencing |
|  | *NEAT1* | 5' GGAGGGAAGTGTTTTTTGTAGAGAT 3' | Forward |
|  |  | 5' ATACCATAAAAAAAAAAACCCCTTTTC 3' | 5’ biotinylated reverse |
|  |  | 5' GTTTGGAATTTTTTAGAT 3' | Forward/  Pyrosequencing |
|  | *PVT1* | 5' TTTTTTAGTAGGAAAGTGGGAAGA 3' | Forward |
|  |  | 5' ATACCCCCCTCAACCTACTTCC 3' | 5’ biotinylated reverse |
|  |  | 5' GGAAAGTGGGAAGAT 3' | Forward/  Pyrosequencing |
